# New encoding concepts for shape recognition are needed

**DOI:** 10.3934/Neuroscience.2018.3.162

**Published:** 2018-07-01

**Authors:** Ernest Greene

**Affiliations:** Laboratory for Neurometric Research, Department of Psychology, University of Southern California, Los Angeles, California, USA

**Keywords:** shape encoding, orientation filters, connectionist models

## Abstract

Models designed to explain how shapes are perceived and stored by the nervous system commonly emphasize encoding of contour features, especially orientation, curvature, and linear extent. A number of experiments from my laboratory provide evidence that contours deliver a multitude of location markers, and shapes can be identified when relatively few of the markers are displayed. The emphasis on filtering for orientation and other contour features has directed attention away from full and effective examination of how the location information is registered and used for summarizing shapes. Neural network (connectionist) models try to deal with location information by modifying linkage among neuronal populations through training trials. Connections that are initially diffuse and not useful in achieving recognition get eliminated or changed in strength, resulting in selective response to a given shape. But results from my laboratory, reviewed here, demonstrate that unknown shapes that are displayed only once can be identified using a matching task. These findings show that our visual system can immediately encode shape information with no requirement for training trials. This encoding might be accomplished by neuronal circuits in the retina.

## 1. Excessive emphasis on orientation-selective filters

The great obstacle to discovering the shape of the Earth, the continents and the oceans, was not ignorance but the illusion of knowledge. Daniel J. Boorstein [1].

A major goal of neuroscience is to explain how image information provides for alternative actions that contribute to survival. There are countless discussions of image “encoding” that attempt to delineate the mechanisms by which neurons extract and summarize the essential information. But “information” can mean a large range of different things, so mechanisms that might “encode” that information would likely have a range that is even larger.

**Figure 1.**
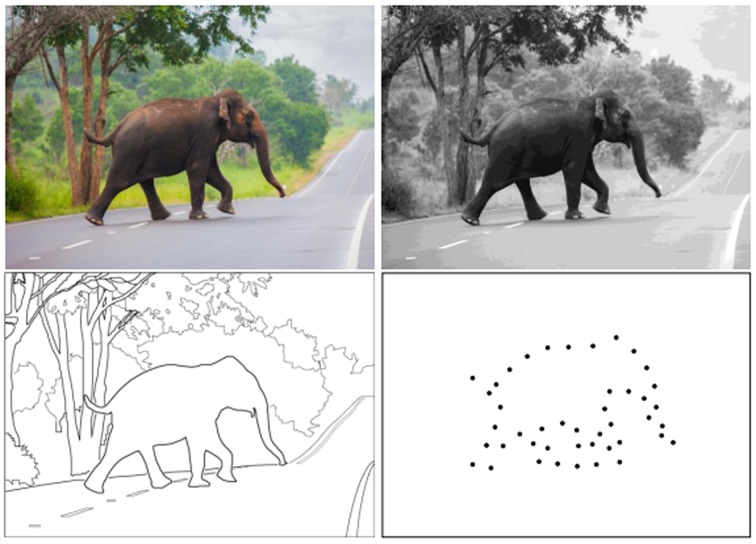
A colored photograph will provide visual cues that can contribute to recognition of objects, but our visual system is generally able to identify an object even if color is eliminated, or if only the outline boundary is provided. Even a sparse array of dots that mark the boundary may be sufficient for recognition of the object's shape. Most models of shape recognition call for registering contour orientation, but the dots in the fourth panel do not provide that information.

To narrow the focus, let's examine which cues are most critical for object recognition, beginning with the color photograph shown in Figure 1. Using a photograph already reduces the complexity of the task, for the real-world scene would have a third dimension. Further, the photograph does not convey changes that might occur in the scene over time, so a contribution of motion is not initially considered.

Now ask the question: “What animal is shown in the scene?” Perhaps, knowing that animals are not green, the neural system would ignore green zones in the image, putting the focus on non-green objects. But the black-and-white photograph that is shown in second panel provides abundant cues that allow for recognition, so it is not fair to say that the non-green color of the elephant is an essential cue for recognition. While not denying that color information can contribute to recognition, most objects can be identified without benefit of color.

Focusing now on the information provided by the black-and-white photograph in the second panel, are the differential shades of gray essential for identifying the animal? Clearly they are not. The scene can be rendered with fine lines that mark the boundaries of objects, as shown in the third panel of Figure 1. Here even the internal contours have been eliminated, leaving only the outer boundaries, yet we can still name the animal figure based on the boundary information. This will come as no surprise to anyone. Artists have long used outline drawings for rendering namable objects; the earliest known examples were scratched on cave walls by our pre-historic ancestors.

Given the fact that fine lines, i.e., contours, can provide sufficient cues for identifying most objects, one can understand why they would be viewed as elemental. Hubel & Wiesel's discovery [2],[3] that neurons in primary visual cortex are especially responsive to the orientation of elongated bars provided the basis for specifying a neural mechanism for shape encoding. One might suppose that successive locations on the shape boundary activate specific neurons that are “tuned” to the orientation at each location, perhaps adding selectivity based on amount of curvature and length. The specific combination of neurons thus activated would therefore specify the shape being displayed.

The fourth panel of Figure 1 serves as a challenge to the orientation-filter concept, for it illustrates that shapes can be identified when the spacing between dots is greater than the length of the receptive fields of orientation-selective neurons. It is well understood that the orientation-selective neurons have a center/surround design wherein the spatial extent of the stimulus will determine whether the cell will be activated or suppressed. An optimal stimulus should have an elongated structure that stimulates the excitatory center without influencing the inhibitory surround, which is substantially longer. Shapley and associates [4] have studied the size of these receptive fields in Macaque monkeys, which have visual systems comparable to that of humans. They examined the lengths of excitatory receptive fields of 50 neurons and found them to have a mean length of 0.82 degrees of visual angle (arc°). None of them had a length greater than 2.0 arc°.

Let's assume that the discrete boundary markers in the fourth panel of Figure 1 are being viewed at the actual scale of the object to be identified. If one assumes the height of the elephant to be 3 meters, at a viewing distance of 10 meters the proximity of adjacent dots would be about 2.5 arc°. This would mean that a given receptive field would be activated by a stimulus that had no orientation nor any other contour information that often has been cited as essential for characterizing shapes. Further, since inhibitory surrounds of orientation-selective neurons range up to 5 arc° [4], neighbors to a given dot would more likely suppress responding than contribute to activation. Finding that shapes can be identified at very low dot densities challenges the concept that contour attributes such as orientation, curvature, and length are essential cues for shape recognition.

**Figure 2.**
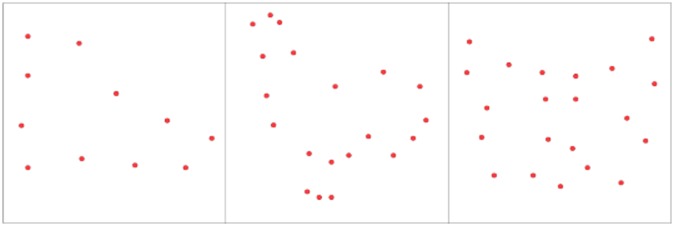
Some shapes can be identified when only a few dots are used to mark the boundary. An inventory of diverse shapes was displayed on a 64 × 64 array of LEDs, wherein dot diameter and span between dots was 4.9 and 9.2 minutes of arc, respectively. A boundary formed by lighting a continuous string of neighboring dots is described as having 100% density. For display at a lower density, only some of the dots were lit. Respondents could identify the boot, rooster, and moth with display of 6%, 7% and 8% of the boundary dots, respectively. Numerous other shapes were recognized when only a small fraction of the boundary dots were shown [5].

As further support of this point, a shape that was displayed with the same low density of dots as in Figure 1 was identified as an elephant by 75% of observers [5],[6]. Many other shapes were recognized by respondents with even fewer dots being displayed, as illustrated in Figure 2. These and related experiments were conducted using custom-designed LED display boards that provided precise control of flash durations and timing. These boards can display a given pattern of dots, including the boundaries of namable shapes, in any order as brief flashes. By flashing all the boundary markers at the same moment, one can preclude any encoding mechanism requiring eye movements. Shapes can be identified with simultaneous flash of all the dots with a duration as brief as one microsecond [7],[8].

One can further test whether contour attributes are critical by comparing dot sequences that should have a differential ability to activate orientation-selective neurons [9]. That experiment delivered the dots forming shape boundaries as successive four-dot subsets with all the dots in a given subset being flashed simultaneously. The dots of a given subset were either an adjacent string of dots or were at random locations on the boundary. The former would provide more effective stimulation of orientation-selective neurons, whereas the latter would not. The probability of shape recognition was essentially the same for these two conditions, further affirming that information about orientation is not a critical shape cue [9].

## 2. Current models inadequately explain how marker locations are encoded

I am asserting that the relative locations of boundary markers provide critical information for shape recognition, and local contour attributes are substantially less important. It is convenient to describe the essential shape cues as boundary markers. It is assumed that edges and lines provide an abundant number of markers, most of which are redundant. Some percentage of markers will be needed to elicit recognition of a given shape with that quantity being different for each shape. The quantity of markers may be as low as three, which provides the perception of a triangular shape [10].

To deal with global positioning of shape elements, quantitative models often just assume a coordinate system that can specify the location of filter elements using addresses. However, there is no evidence that the nervous system can or does provide address values. Neural network (connectionist) models bypass the need for addresses by adjusting the strength of connections to achieve non-local summaries of shapes, but do so at a cost.

Neural network models embrace the concept that global positioning of stimulus elements can be rendered moot by tailoring the connections among successive neuron populations. Shape encoding is based on modification of connections that are initially diffuse. Fukushima's Neurocognitron [11] was one of the earliest models and was designed to identify printed text. It was inspired by the neurophysiological work of Hubel & Wiesel [2],[3], and the computational elements were viewed as functionally equivalent to neurons. The model contained several layers, with the neurons of a given layer being connected by communication links to neurons in the next layer. The function of simple (S) neurons was to register local patterns of activation provided by each letter, with filtering for orientation of the strokes being a major task. That information would pass through the communication links to complex (C) neurons, which registered combinations of the local patterns. His model had each neuron in one layer being connected to many neurons in the next layer. The initial diffuse connectivity would not produce any recognition of specific patterns of activation in the sensory layer. However, training trials produced successive changes of connection strength that eventually provided selective activation of one or more neurons in the output layer in response to a given stimulus shape or pattern. After extensive training, only one or a few neurons in the final layer would respond to a specific letter irrespective of where it was displayed on the sensor array. Numerous variations of the basic concept have been formulated. Most notable are the works of Rolls [12],[13], Rodriguez-Sanchez & Tsotsos [14], Riesenhuber & Poggio [15], Pasupathy & Connor [16], Suzuki *et al.* [17], and Pinto & associates [18].

All connectionist models require training to provide for encoding and recognition of shapes, and even more training trials are needed to deal with translation, rotation, or changes in size. An effective model needs to develop a pattern of synaptic strength that will provide for recognition of a given shape irrespective of where it is displayed on the input layer, or is shown as rotated or in various sizes. A given shape cannot be recognized unless the training has brought about the right balance of influence across the many connections within the network.

**Figure 3.**
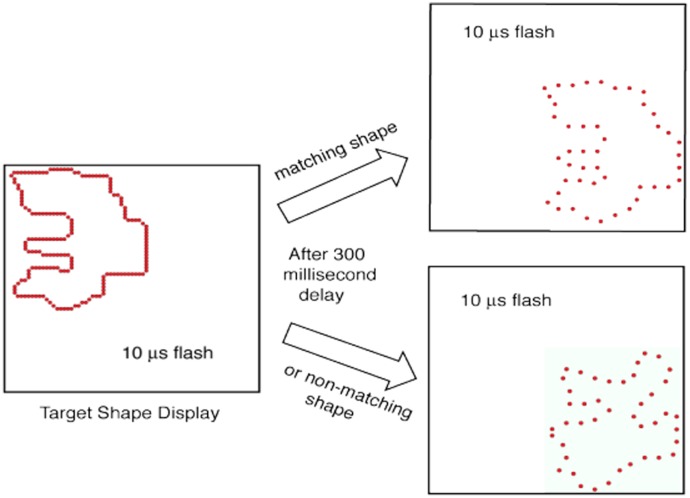
One can focus on the shape-encoding process by using shapes that are not stored in long-term memory. The matching task illustrated here displays an unknown shape as a target only once, followed quickly by a comparison shape that is either a low-density version of the same shape or was derived from a different shape. Data analysis can correct for response bias using signal-detection theory. Display location can be varied to demonstrate translation invariance. One can modify the size or orientation of the comparison shape to provide evidence of size and rotation invariance.

Recent work from my laboratory challenges connectionist models by providing evidence that an unknown shape that is seen only once can be identified a moment later using a match protocol [19]. The task conditions are illustrated in Figure 3. It called for a brief display of a target shape that was quickly followed by a low-density comparison shape that was the same as the target or was different. Respondents verbally rendered a same/different judgment. The shapes were drawn from an inventory without replacement. Displaying an unknown shape only once and asking for an immediate decision about whether the comparison was the same shape eliminates any training requirement or use of long-term memory. It is likely that working memory preserved the shape information. To avoid any confusion about the basis for shape identification, it is convenient to describe the judgments that were required in this task as “match recognition”.

**Figure 4.**
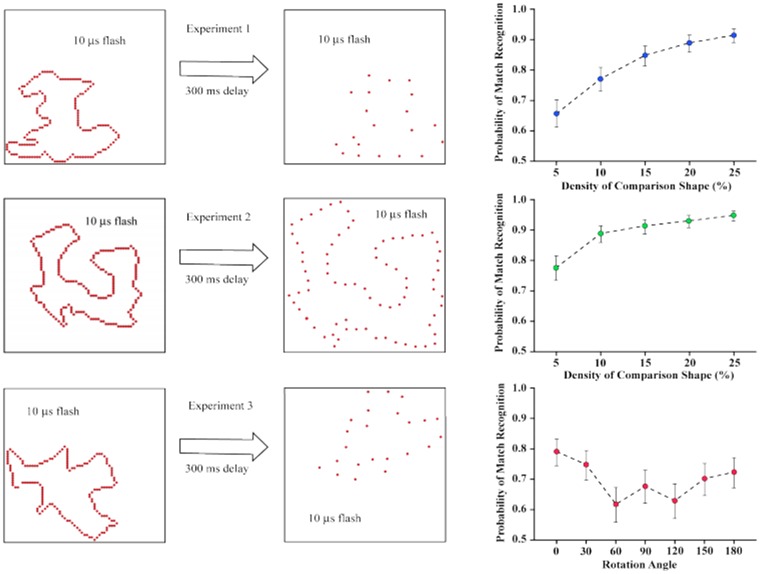
Each of the three experiments [19] displayed an unknown shape as a target only once at 100% density. Only matching shapes at 12% density are illustrated here, but a non-matching shape was displayed on half of the trials. Experiment 1 varied density of the comparison shapes; match recognition remained well above chance even when these shapes were displayed at very low densities. Experiment 2 displayed the comparison shapes at an enlarged size and demonstrated that the encoded shape summary was size invariant. Experiment 3 varied the orientation of the comparison shape relative to the target, demonstrating rotation invariance.

The match recognition task has provided a number of interesting results, with some being illustrated in Figure 4. One experiment [19] found that match-recognition probability was above 0.90 with a comparison-shape density of 25%. The probability declined as density was reduced, but was still well above chance with a 5% density. The target and comparison shapes were shown in different corners of the display board, thus demonstrating that the encoding mechanisms that accomplished match recognition were translation invariant. Similar effects were found in a follow-up experiment [20] that used 20% and 4% densities for the target shapes, and with comparison shape densities ranging from 20% to 4%.

Responses were evaluated with signal detection analysis that derived an unbiased index of performance—*p(c)*_max_—which allowed conclusions about whether the probability of a correct response was above chance. For convenience, the present discourse will describe this index as “probability of match recognition”.

The second experiment illustrated in Figure 4 used comparison shapes that were much larger than the targets, with both being displayed at the center of the board. Match recognition declined less as a function of density, clearly affirming that the encoded summary was size invariant. All comparison shapes for Experiment 3 were at 12% density. For trials that displayed a comparison shape that matched the target, its orientation was varied across a range from 0° to 180°. The probability of match recognition remained well above chance at all orientations; this demonstrated rotation invariance.

**Figure 5.**
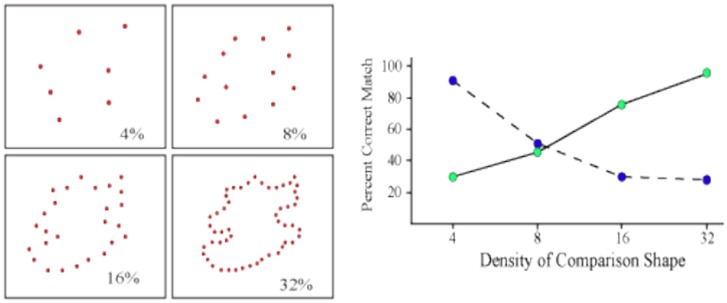
The experiment reported in [21] displayed target shapes at 4% (blue plot points) or 32% density (green plot points), and varied the density of comparison shapes at 4, 8, 16 or 32% density. The percent of correct matches declined as a function of the size of the difference between target and comparison density.

One might think that a decline in match recognition as density is reduced is a simple matter on how much information each dot contributes to the encoding process. Fewer boundary markers might deliver less shape information and thus lower the ability to register shape attributes and use them for shape identification. That interpretation is belied by a simple experiment [21] that used targets at either 4% density or 32% density, and varied the density of comparison shapes across the range from 4% to 32%—see Figure 5. For the 32% targets, there was a decline in identification of matching shapes as their density was reduced from 32% to 4%. For the 4% targets, the percentage of correct matches was maximal when the comparison shape was at 4% density, and it dropped as comparison-shape density increased. In other words, the 4% targets were fully capable of providing shape information that put match recognition in the 90%–100% range. The shape summary that was encoded from this low quantity of target dots was undermined as the number of dots in the comparison shape was increased. The low-density target suggested one shape and the higher-density comparison shape suggested something different.

The results shown in Figure 5 is at odds with the simple concept that the higher the density of the dots, the more shape information is conveyed by the stimulus. Rather, a shape is implied even when the target contains very few dots, and adding dots can reduce the degree to which the comparison matches that shape summary.

More generally, the ability to encode and identify these unknown shapes at very low densities reinforces the point that orientation information is not essential for shape recognition. The longest receptive field span of orientation-selective neurons is around 2 arc° [4], so the orientation of adjacent dots will not be registered if the separation is greater. For the 4% condition illustrated in Figure 5, about 40% of the matching shapes had all dots separated by at least 2 arc°, yet identification was roughly the same as for all the shapes.

Connectionist models, especially those based on deep neural networks, have proven to be effective at identifying objects that are shown in snapshots or in videos. However, much of the motivation for their development came from claims that they simulate brain mechanisms. Human visual skills are vastly superior to the best computational models that have been crafted thus far. Whether further improvement of the connectionist concept will eventually match our abilities is an open question. I think not. It seems likely that an entirely different kind of encoding mechanism makes it possible to see an elephant in the dot pattern shown in Figure 1, and provides for identification of an unknown shape even if it is translated, rotated, or altered in size [22].

## 3. New concepts are needed to explain the encoding of global position information

**Figure 6.**
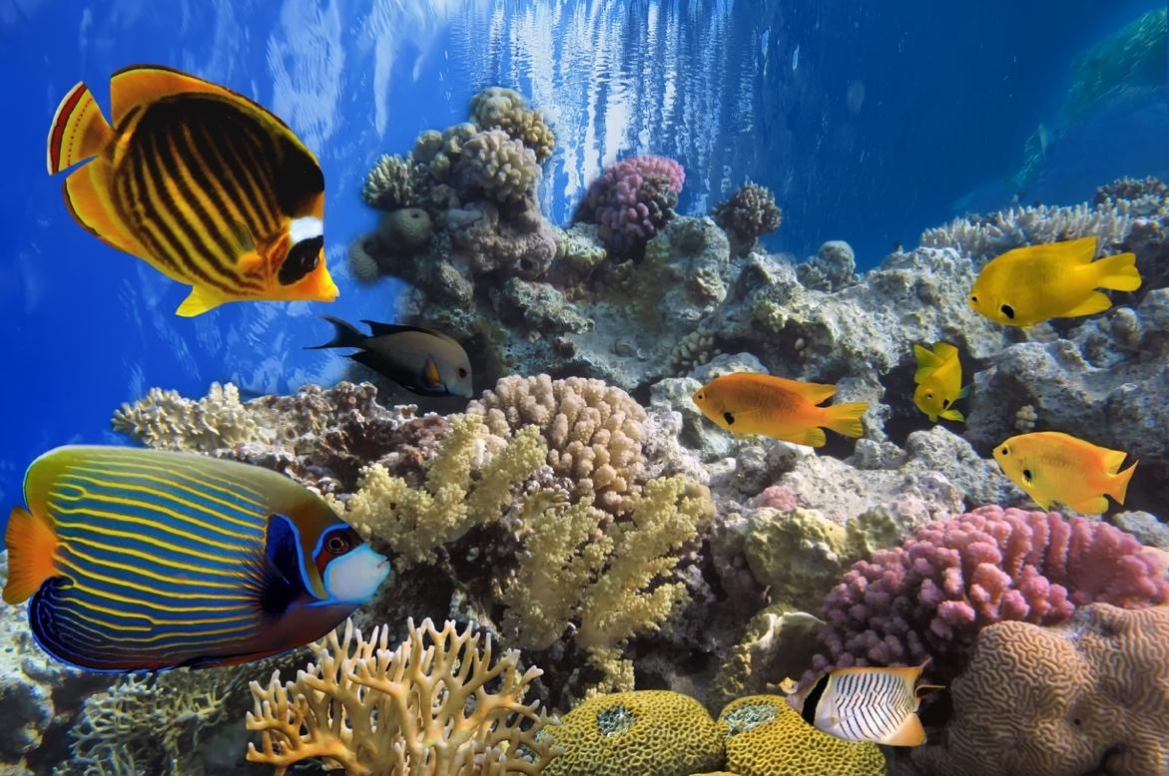
The visual system of fish provides for recognition of shapes that are critical to their survival. They must process complex scenes that include sources of food and places to hide. They must identify potential predators to avoid being eaten and recognize the shapes and patterns of their own species to find potential mates.

It troubles me that the connectionist models are so heavily focused on cortical neurophysiology. Do we think that species with smaller nervous systems have minimal ability to encode shapes? Fish can identify shape cues that contribute to their survival. The photograph in Figure 6 illustrates that fish must process the complex shapes of rocks and coral that provide food and shelter. Clearly they can also identify potential predators and members of their own species that are suitable mates. A number of experimental studies have confirmed some of these skills [23]–[28].

The visual system of fish mainly consists of the retina and optic tectum, the latter being known in mammals as the superior colliculus. This raises the intriguing possibility that elemental shape encoding, especially the encoding of global positioning of boundary markers, is being done in the retina or possibly the superior colliculus. There are some substantial benefits to this concept. For one, it could avoid the strange paradox of having components of the shape being sent to different hemispheres, which occurs in our visual system through ipsilateral and contralateral optic-nerve fibers from nasal and temporal hemiretina. The anatomy of early layers within the retina have continuous tiling that does not functionally split the scene into two halves, as occurs once the signal passes into the optic nerve. It seems reasonable that relative locations of boundary markers would be processed by neural circuits that can integrate both halves of an object at an early stage of encoding (see reference 10 for additional discussion of this point).

Another source of intuitions about shape encoding was provided by interactions with a young mathematician, Peter Waksman, who convinced me of the benefits of summarizing 2D shapes as 1D functions. Whereas the means to compare mismatch of boundary contours or markers that are arrayed in 2D space can be complicated, there are simple methods for comparing 1D functions that can specify their similarity. The collaboration with Waksman produced a paper and a patent [29],[30], neither being concerned with biological plausibility of the concepts. The goal was simply to establish that a 1D summary could be effective for recognizing shapes.

For dealing with discrete boundary markers, one might use a distribution of distances from markers to the centroid [10],[19],[31],[32]. The distance to centroid concept is biologically plausible if one assumes a retinal encoding mechanism wherein the stimulated locations generate spreading waves that converge to be maximal at the centroid. These distance-encoding waves might be provided by polyaxonal amacrine cells of the retina.

Polyaxonal amacrine cells were first characterized in primate retina [33]. They have a narrow dendritic receptive field that would provide good resolution for registering contour markers. However, unlike other amacrine cells that lack axons, the polyaxonal amacrine cells have axonal arbors that branch in all directions [33]–[40]. In the Macaque, where these cells were first described, the axons extend across roughly ten times the area of the receptive field [33]. The span, when converted to the dimensions of human retina, would cover about 7.5 arc°. When a polyaxonal amacrine cell is stimulated, spikes spread like a ring through the axon arbor [35],[38],[39]. Wright & Vaney [40] report that they connect to a single class of retinal ganglion cell, these being “local edge detectors”.

As discussed in earlier papers [10],[41], the simultaneous display of boundary markers might activate one or more polyaxonal amacrine cells at each location, providing spreading waves that reach the centroid at different times. Retinal ganglion cells located at the centroid would register a complex waveform, providing the functional equivalent of a distribution of marker-to-centroid distances.

Note that this particular centroid-based concept has ignored the angle at which a given marker lies, and provides only the functional equivalent of a distribution of marker-to-centroid distances. One might think that this would provide a very weak basis for identifying shapes, but even without the angle information, the method is surprisingly effective. With summaries derived from hundreds of shapes, one will typically find that none are misidentified when each shape is paired with every other shape in the inventory [31]. Further, one can ask for recognition of a shape that has been substantially reduced in the number of boundary markers, and still have very few recognition errors [31].

**Figure 7.**
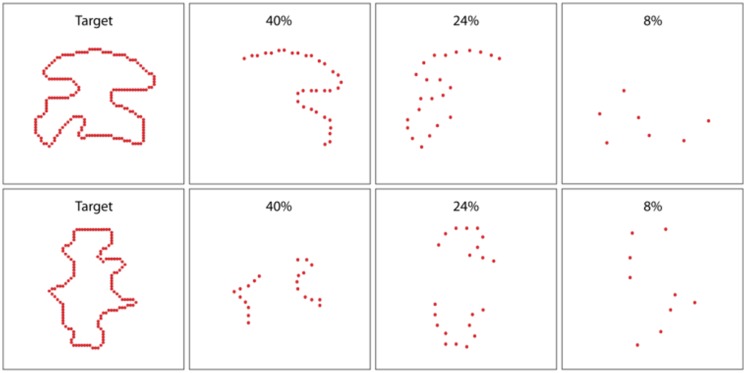
The upper panels show an experiment [from reference 42] wherein the unknown shape was shown as a target at 100% density, and comparison shapes were displayed at densities that ranged from 8% to 40%. Further, comparison shapes displayed only half of the boundary perimeter, with the bisection angle being chosen at random. Match recognition declined as a function of density, but remained significantly above chance across the full density range. The lower panels show similar task condition, except two portions of the boundary, each being one-quarter of the perimeter, were provided on opposite sides of the shape. The bisection condition of the upper panels would yield centroids at a different locations than the targets, whereas the centroids of comparison shapes in the lower panels would be closer. Both conditions produced match-recognition results that were almost identical.

Our laboratory has, however, found evidence that is at odds with the concept that shapes are summarized using marker-to-centroid distances. Nordberg et al. [42] evaluated match recognition wherein the targets were displayed at 100% and comparison shapes were displayed at a lower density and with deletion of portions of the boundary perimeter—see upper panels of Figure 7. With half of the boundary perimeter being deleted, randomly varying which half, respondents provided above-chance match recognition at the lowest density that was tested, this being 8%. This results would not be predicted for a summary method that was centroid based. Where only half of the perimeter contains markers, the centroid of the comparison shape would differ from its location for the target, thus any summary based on the centroid-distance measures would be different.

A companion experiment examined match recognition of comparison shapes wherein portions of the boundary were displayed on opposite sides of the centroid, i.e., a quarter of the perimeter on each side. This configuration would provide a centroid for each comparison shape that would be very close to the centroid of its target. If a centroid-based summary were being generated, match recognition should be much higher for this condition than for the experiment that used non-symmetric boundary markers. Yet the plots of match-recognition for symmetric marker and non-symmetric markers were almost identical [42].

As an alternative encoding hypothesis, Nordberg et al. [42] suggest that the relative positioning of markers is registered using scan waves. Here we have adopted the suggestions of Bullock [43], Hopfield [44], as well as Thorpe and associates [45]–[48], who proposed encoding of stimulus attributes based on time-to-fire of early sensory neurons. The basic concept is that essential, stimulus-defining information is conveyed by the initial wave of spikes that are generated across a population of neurons, rather than in the frequency of firing of individual neurons. Some have called this a “population code”. Further adaptation by Nordberg et al. [42] assumes that marker-stimulated locations do not produce spikes until a polling wave crosses each location. Polling waves might be generated by polyaxonal amacrine cells. As a polling wave spreads across the retinal array, each marked location generates a spike, such that the overall population response consists of varying levels of spike density being delivered by the optic nerve. The proposed mechanism for shape encoding can be designated as “scan encoding”.

The use of scan waves to encode shape might have evolved from motion-activated mechanisms. Several teams of investigators have suggested that motion plays a role in the encoding of shape information. Ahissar & Arieli [49] propose that small eye movements during fixation convert shape contours into a temporal code. Rucci & Victor [50] provide evidence that fixational drift improves the visibility of high-frequency spatial gratings. Gollisch & Meister [51] propose that synchronous firing of retinal ganglion cells is triggered at the end of a saccadic eye movement. Such synchronous firing may provide a population response that registers contours.

A recent experiment from my laboratory appears to support a scan-encoding concept [52]. Here each unknown shape was scanned to produce histograms of the number of dots encountered. Specifically, for each shape the number of dots in each row and in each column of the display board were counted, providing a single 128-bin histogram. The histogram was then trimmed of empty bins, i.e., deleting empty rows and columns and including only those that contained boundary dots. The resulting histograms were re-binned to provide a 20-bin histogram for each shape, and were normalized to provide bin heights that summed to 1.0.

**Figure 8.**
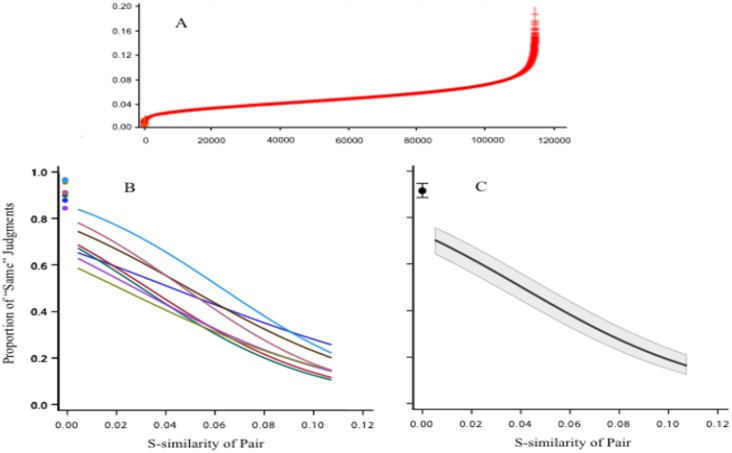
A: Histograms derived from the 480 unknown shapes were paired, and a sum-of-squared-differences value was derived for each pair. This plot of the ranked values provides a scale for specifying the degree of similarity of the pair members. B: Three-hundred of the pairs were tested using the match-recognition task, and the regression model for each of the respondents is plotted. Each manifested a near linear decline in match-recognition as a function of the similarity value. Mean judgments for trials where the target and comparison were the same shape are shown with dots at the scale value of zero. C: The mean regression model for the group of respondents is plotted, along with the 95% confidence interval for the model [52].

Each shape in the inventory was then paired with each other shape to derive an index of similarity for each pair. For the inventory of 480 shapes, this provided 114,960 pairs. An index of similarity was derived by comparing the pair histograms using a sum-of-squared-differences calculation. These scan-similarity values were then ranked for size; a plot of the ranked values can be seen in Figure 8A. There were no duplications of the similarity value, so the histograms derived using the scan method would provide an unambiguous basis for identifying each shape.

Three-hundred pairs were chosen at equal intervals across the ranked similarity values. These were displayed to respondents in the match task. One member of the pair served as the target and the other as the comparison shape. Ninety additional shapes were added wherein the same shape was used both as target and comparison shape. As in several of the experiments described above, the targets were displayed at 100% density and the comparison shapes were displayed at 12% density. Providing a degraded stimulus was needed to prevent discrimination of small differences.

The size of the scan-similarity value predicted the probability of match judgment, as shown in Figure 8B and 8C. With values that reflected high pair similarity, the probability of match judgment was about 0.7. The probability declined at a near-linear rate as the size of the scan-similarity value increased, averaging only about 0.2 probability for the highest values.

These results affirm that the scan-encoding concept is a potential basis for match recognition. The scale values that are derived by this method can statistically predict the degree to which humans will identify a comparison shape as being the “same” as the target. Additional experiments evaluating alternative scan-encoding methods are underway.

Note that the scan encoding mechanism described above is fundamentally different from the connectionist model. Scan encoding transcribes the shape into a message, which is modeled as a histogram, distribution, or waveform. A population code might be used to deliver the message or it might be sent as wavelet components. The key point is that being encoded as a message is fundamentally different than being encoded as a tailored set of connections or a specific balance of connection strength. Establishing how such messages could be generated, transmitted, stored, and retrieved deserves further thought.

## 4. Final thoughts

**Figure 9.**
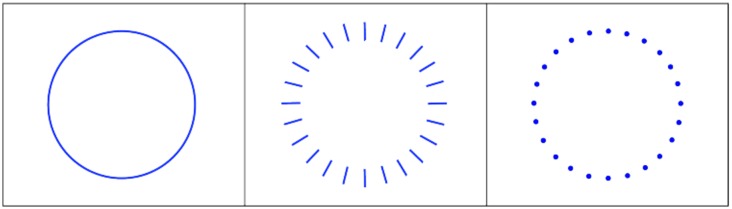
The left panel provides a full complement of cues that characterize a circle. The middle and right panels provide circular patterns, the criterion for that being the relative positioning of elements.

In closing, let's return to the argument that the contribution of orientation-selective neurons has been overemphasized. A simple circle is shown on the left panel of Figure 9. What are the cues that provide it with a circular shape? The middle panel shows a circular array of short segments, with each oriented at 90 degrees from the original contour. The right panel shows each segment being replaced with a dot. In formulating theories of how the circle is encoded, many models have invoked the neurophysiology of Hubel & Wiesel [2],[3], putting the emphasis on the activation of orientation-selective neurons by local segments of the contour. But any activation by the middle and right configurations are at odds with a role for orientation. Are they not circular?

Most would agree that neither is a complete circle but they do form circular patterns. The local orientation of contour components, their curvature, and presence of continuity are clearly relevant to perception and can be pertinent to one's criteria for defining a given shape. In like manner, the presence of color can determine how one classifies a shape, e.g., as a leaf or as a feather. Every feature of the object that can be discriminated can provide a basis for classification and may contribute to recognition. However, recognition of a given object seldom requires the details that are provided by an intact contour.

Emphasizing orientation, curvature, and linear extent obscures the importance of spatial positon as an essential shape cue. It is not clear how spatial position information is derived by the nervous system, or how that position information is used to summarize a given shape, or how that summary is stored in memory. More progress may come if investigators and theorists direct more of their attention to these issues.
